# Response to Commentary on “Reassessing Simultaneous Pancreas Kidney Vs. Kidney Transplant Alone: A Propensity Weighted Analysis of Survival and Morbidity”

**DOI:** 10.3389/ti.2025.16011

**Published:** 2025-12-30

**Authors:** Pooja Budhiraja, Rocio Lopez, Susana Arrigain, Jesse D. Schold

**Affiliations:** 1 Department of Medicine, Mayo Clinic Arizona, Phoenix, AZ, United States; 2 Department of Surgery, University of Colorado Anschutz Medical Campus, Aurora, CO, United States; 3 Department of Epidemiology, University of Colorado Anschutz Medical Campus, Aurora, CO, United States

**Keywords:** ddkt, diabetes mellitus, kidney transplant, SPK transplantation, survival analysis

We thank Drs. Gruessner for their thoughtful and experienced perspectives on our manuscript. We acknowledge that simultaneous pancreas-kidney transplantation (SPKT) provides important metabolic benefits, including insulin independence, improved glycemic control, and possible mitigation of diabetic complications. These advantages remain central to patient and clinician decision-making. Unfortunately, these outcomes are not captured in national registry data, which lack information on insulin use, degree of beta cell failure, cardiovascular status, or microvascular complications. Accordingly, our analysis focused on outcomes that are uniformly recorded for all transplant recipients, including patient survival, kidney graft survival, treated acute rejection, and hospital readmissions.

We agree that differences between SPKT and deceased donor kidney transplant recipients present challenges for direct comparison. This is the reason why we used overlap propensity weighting, a method designed to reduce bias in scenarios with limited overlap. Although residual confounding cannot be fully eliminated in observational studies, this approach helps reduce the influence of donor and recipient characteristics that historically favor SPKT in unadjusted analyses. Many prior studies showing superior survival with SPKT included younger donors, lower Kidney Donor Profile Index kidneys, shorter dialysis times, and overall healthier baseline characteristics in the SPKT cohort. These differences can amplify survival signals, making it difficult to distinguish the effects of pancreas transplantation from other influencing factors.

Our findings reflect contemporary practice, where advances in surgical techniques, immunosuppression, perioperative monitoring, and postoperative care have improved kidney-only transplant outcomes, narrowing historical survival differences. In addition, the landscape of diabetes care has evolved with the wider use of therapies such as glucagon-like peptide-1 receptor agonists and sodium-glucose cotransporter-2 inhibitors, which offer metabolic, cardiovascular, and kidney-protective benefits and are increasingly accessible, particularly among patients with Type 2 diabetes [1, 2]. These advances support individualized SPKT selection, since metabolic improvement may be achievable in some recipients through medical therapy, while transplantation remains most impactful for those with clear insulin deficiency and appropriate surgical risk.

Our subgroup findings provide meaningful context. Among recipients with Type 1 diabetes and BMI below 30, which aligns closely with physiological rationale and traditional listing criteria, SPKT was associated with superior survival. Outside this phenotype, survival was similar, while early morbidity remained higher for SPKT. These findings support careful, individualized selection rather than broad application of SPKT to all diabetic transplant candidates.

We share the authors’ longstanding commitment to ensuring that SPKT remains available to those most likely to benefit. Our intent is not to diminish its role but to provide objective empirical evidence that informs nuanced counseling and allocation practices. Incorporating both metabolic strengths and elevated early clinical risks into shared decision-making encourages balanced patient-centered care. We hope our study contributes to ongoing dialogue and supports thoughtful prioritization of pancreas grafts for candidates who stand to gain the most.

## Data Availability

The original contributions presented in the study are included in the article/supplementary material, further inquiries can be directed to the corresponding author.
